# Should Patients Be Allowed to Pay Out of Pocket? The Ethical Dilemma of Access to Expensive Anti-cancer Treatments in Universal Healthcare Systems: A Dutch Case Study

**DOI:** 10.1007/s11673-024-10342-2

**Published:** 2024-09-26

**Authors:** C. H. C. Bomhof, Eline M. Bunnik

**Affiliations:** Department of Medical Ethics, Philosophy and History of Medicine, Erasmus Medical Center, Wytemaweg 80, 3015 CN Rotterdam, the Netherlands

**Keywords:** Medical ethics, Anti-cancer treatments, Funding and reimbursement, Universal healthcare, Out-of-pocket payments

## Abstract

With the increasing prices of newly approved anti-cancer treatments contributing to rising healthcare costs, healthcare systems are facing complex economic and ethical dilemmas. Especially in countries with universal access and mandatory health insurance, including many European countries, the organizing of funding or reimbursement of expensive new treatments can be challenging. When expensive anti-cancer treatments are deemed safe and effective, but are not (yet) reimbursed, ethical dilemmas arise. In countries with universal healthcare systems, such as the Netherlands, this gives rise to a rather new ethical dilemma: should patients be allowed to pay out of pocket, using private funds, for medical treatments? On the one hand, to allow patients to pay for treatments out of pocket would be in line with the medical-ethical principles of beneficence and autonomy. On the other hand, allowing patients to pay out of pocket for anti-cancer treatments may lead to unequal access to medical treatments and could be considered unfair to patients who are less well-off. Thus, it could undermine the values of equality and solidarity, on which the Dutch healthcare system is built. Furthermore, out-of-pocket payments could potentially lead to financial hardship and distress for patients, which would conflict with the principle of non-maleficence. Does this mean that patients can rightfully be denied access to approved but not (yet) reimbursed anti-cancer treatments? In this article, we will use the Dutch healthcare system, which is based on equal access and solidarity, as a case study to draw attention to this—currently relatively unknown and unresolved—dilemma and to *clarify* the values at stake. This article contributes to current discussions about the societal problem of rising healthcare costs by informing policymakers, healthcare professionals, and ethicists about the ethical dilemma of out-of-pocket payments in universal healthcare systems, and aims to support health authorities, policymakers and health professionals in developing policy for whether to allow out-of-pocket payment-based access to newly approved but (too) expensive anti-cancer treatments.

## Introduction

Because of the rising costs of healthcare, health authorities and policymakers worldwide are facing complex economic and ethical dilemmas. Especially in countries with universal healthcare systems, including many other European countries, the organizing of public funding of expensive new anti-cancer treatments can be challenging. Newly approved anti-cancer treatments, such as immunotherapies and cell therapies, can cost tens of thousands or even hundreds of thousands of euros per patient, respectively (Evernorth Research Institute. 2023; Hernandez et al. 2018). When anti-cancer treatments are approved for marketing but—because of the costs—(temporarily) not made available through the universal healthcare system, patients are often denied access to such treatments, as out-of-pocket payments by patients themselves, using private funds, is not common, not deemed acceptable, or not even possible in many countries. Policymakers, hospital boards, and the medical profession are confronted with an ethical dilemma: should patients be allowed to pay out of pocket for—safe and effective—non-reimbursed anti-cancer treatments?

Until now, ethical analysis of out-of-pocket payment in universal healthcare systems has been lacking in the literature. While there has been extensive ethical and philosophical debate on fair allocation of resources, limit setting, and distributive justice in healthcare (Callahan 1987; Daniels 2001; Ehni 2014), most theories focus on the fair allocation of collective funds, not on the fairness of individual patients’ private spending on healthcare services. Thus, most theories of health justice might conclude that to sustain publicly funded healthcare systems, healthcare expenditures must be contained, and resources should be allocated only to health services and medical treatments that meet agreed-upon cost-effectiveness requirements. Therefore, it may be just for a publicly funded healthcare system to decide against reimbursement of newly approved anti-cancer treatments that are “too expensive” or that are of unknown or uncertain cost-effectiveness. Consequently, it may be just for such a healthcare system to deny coverage for—and therewith, to hinder patient access to—these treatments. However, these theories often refrain from addressing the question whether or not it is just to allow patients who can afford to pay for the treatment themselves, to do so.

To our knowledge, the problem of out-of-pocket payment for non-reimbursed treatments in universal healthcare systems, has not yet been extensively assessed from an ethical perspective. Researchers have addressed ethical aspects of top-up fees, private payments, or co-payments for cancer care in countries with universal healthcare systems, such as New Zealand, the United Kingdom, and Sweden (Färdow et al. 2019; Fenton 2011; Sikora and James 2009; Bloor 2008; Gubb 2008; Richards M. 2008). However, some of these countries also have parallel (small) private healthcare sectors. In the Netherlands, there is no such private sector. As in the Netherlands, it is not deemed socially acceptable to have patients pay for medical treatments out of pocket, there is currently very little opportunity for patients to get access to newly approved but non-reimbursed anti-cancer treatments at all. The Dutch healthcare system is profoundly based on the values of equal access to healthcare and solidarity (see textbox 1), and allowing private spending on medical treatments would be considered diametrically opposed to the underlying ethical values of the healthcare system. In this article, we would therefore like to draw attention to this highly urgent dilemma of allowing out-of-pocket systems by using the Dutch healthcare system as a case study to discuss the question: is it fair to withhold access to newly approved anti-cancer treatments from patients who may be able to pay for these treatments themselves?

For some readers, notably in countries with two-tier healthcare systems or with systems with large private sectors, it may be difficult to imagine that—and on what grounds—patients would be denied access to approved medical treatments, or it may seem self-evident that patients’ liberty rights would trump any justice-based arguments against out-of-pocket payments. Therefore, the aim of this article is to provide insight in the ethical dilemma posed by out-of-pocket payments for expensive anti-cancer treatments in the context of a country with a universal healthcare system, and insight into the arguments both for and against allowing out-of-pocket payments. With the expected regulatory approval of many new expensive anti-cancer treatments in the near future, clarification of the ethical stakes of the problem of out-of-pocket payments is urgently needed. This article is meant to help raise awareness among policymakers and contribute to the design of suitable courses of action for the provision of access to newly approved expensive anti-cancer treatments.

## Case Study: Out-of-Pocket Payments in the Dutch Healthcare System

In 2018, the Dutch National Institute for Public Health and the Environment (RIVM) predicted that by 2040 the healthcare costs in the Netherlands will have increased by twofold. Of all diseases, the costs of treatment of cancer will increase the most, around 6 per cent per year, mainly because of newly approved expensive oncological treatments (Dutch National Institute for Public Health and the Environment 2018). Traditionally, in the Netherlands, all newly approved in-hospital drugs have been automatically and immediately reimbursed within the “basic package” of national health insurance, for all patients (see Textbox 1). However, in 2015, the Dutch government introduced a new policy measure to curtail the costs of expensive newly approved treatments. Nowadays, all new in-hospital treatments which enter the market and are expected to cost more than 50,000 euro per patient per year with a total budget impact of ten million euro, or which are expected to have a total budget impact of twenty million euro for all clinical indications, are put on hold in a so-called “Coverage Lock” (Zorginstituut Nederland 2021; Kuipers 2023). While treatments are in the Coverage Lock, they are not reimbursed (yet) within the national health insurance. In the meantime, the Dutch National Health Care Institute develops advice on the cost-effectiveness of the treatment while the government negotiates with the manufacturer in an attempt to lower the price. Since 2015, 57 treatments have been placed in the coverage lock (Zorginstituut Nederland 2023). The estimated average time which treatments spent in the coverage lock currently is 587 days (Vereniging Innovatieve Geneesmiddelen 2023). However, there is no formal limit to the duration of time that treatments can be put on hold in the Coverage Lock. While treatments are in the coverage lock, patient access is limited. In some cases, pharmaceutical companies cover the costs of the treatment while a reimbursement decision is pending. However, this is not always the case. As a consequence, patients do not always have access to newly approved treatments while these are in the coverage lock. This is a relatively new phenomenon in the Netherlands: medical treatments which have entered the market and are considered safe and effective are not (yet) made available to patients, because of their costs or uncertainty about their cost-effectiveness.

Dutch physicians tend not to prescribe medical treatments that are not (yet) reimbursed through the national healthcare system (Bomhof et al. 2022). On the one hand, physicians may wish to prescribe such treatments to improve patient health and to respect the autonomy or liberty of patients and allow them to spend any private funds as they see fit. Yet on the other hand, physicians may be reticent about allowing patients to pay out of pocket, as they may not want to introduce or exacerbate inequalities in access to healthcare. Legally, in the Netherlands, when treatments are approved for marketing by the European Medicines Agency (EMA) or the Dutch Medicines Evaluation Board, doctors can prescribe them. While, in theory, patients could pay for treatments themselves, it seems that out-of-pocket payments for treatments are not common in our country (Calcoen 2017). Little is known about in-hospital practices or about whether out-of-pocket payments are allowed according to local hospital regulations in the Netherlands. To our knowledge, there is no national policy regarding out-of-pocket payments. As a result, individual physicians and/or hospitals are likely handling access to and funding of non-reimbursed treatments differently. Such discrepancies in practices amongst physicians and hospitals are undesirable and may lead to societal unrest. Therefore, it is important that hospital administrators, policymakers, and representatives of the medical professions are aware of the problem of out-of-pocket payments and design policies to harmonize practices and take away moral uncertainty from the consultation room.

**Textbox 1:**
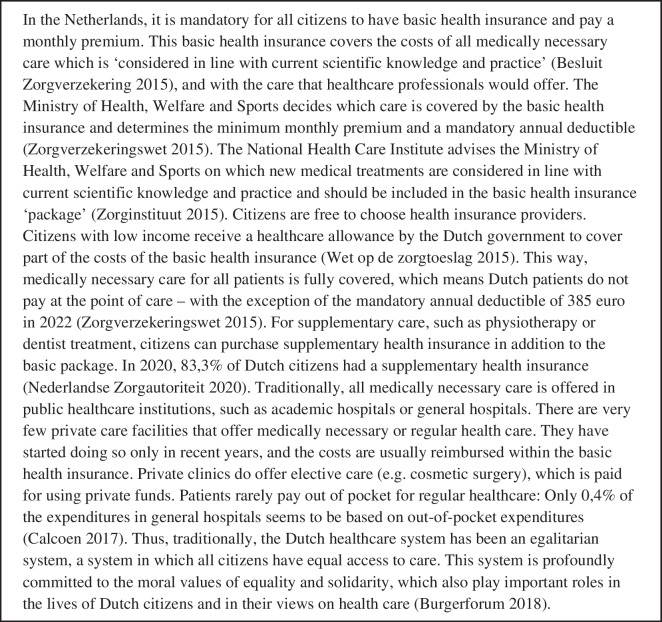
the Dutch healthcare system

## Ethical Arguments in Favour of Out-of-Pocket Payment

In this article we consider treatments which are approved for marketing by the relevant drug regulatory authority (e.g. EMA), and so have been proven safe and effective based on clinical trials but are not (yet) reimbursed through the healthcare system, either because reimbursement decision is still pending, such as for treatments in the Coverage Lock, or because they have not been deemed eligible for reimbursement at all due to limited cost-effectiveness. Two main arguments in favour of allowing patients to pay for treatments out of pocket, we believe, are evident.

### Beneficence

At first glance, the most obvious argument to allow out-of-pocket payment is the possible medical benefit it could bring for patients. If a treatment is not provided by the publicly funded healthcare system, out-of-pocket payment may be the only way for patients to access the treatment and its associated benefits. Allowing patients to pay and improve their health would be in line with the value of beneficence. Beneficence is one of the oldest moral values in medicine and was already mentioned in the Hippocratic oath around 400BC. In their foundational book of 1979, Beauchamp and Childress presented beneficence as one of four principles of biomedical ethics, together with respect for autonomy, justice, and non-maleficence, which will be discussed below. Together these four principles express the basic moral commitments of the doctor–patient relationship by which physicians are bound and play a central role in contemporary medical ethics (Beauchamp and Childress 1979). Beauchamp and Childress defined beneficence as an act of mercy, kindness, and charity (Beauchamp and Childress 1979). In medical ethics, beneficence is often considered to be the duty of healthcare professionals to act in the best interest of the patient, to promote well-being, to improve health, to alleviate suffering, and to prevent harm.

It should be noted that the actual benefits of newly approved anti-cancer treatments might sometimes be limited or even marginal. In a retrospective study, Davis and colleagues showed that of the newly approved anti-cancer treatments which had entered the global market between 2009 and 2013, only 10 per cent had an effect on the improvement of quality of life and only 35 per cent established a significant prolongation of survival. They also found that the magnitude of the benefit on overall survival ranged from 1.0–5.8 months (Davis et al. 2017). In another study, Grössman and colleagues showed that the majority of the therapies which were approved by the EMA between 2009 and 2016 had a survival benefit of less than three months (Grossmann and Wild 2016). In a follow-up study they showed that one-third of oncology drugs with ambiguous benefit-risk profiles at the time of approval, eventually failed to demonstrate a survival benefit several years after market authorization (Grossmann et al. 2019). However, sometimes expected effects might be significant, for instance in the case of new immunotherapies such as Yescarta, a form of CAR-T cell therapy which costs around 400,000 euros per patient and is potentially curative (Hernandez et al. 2018). Hence, while the benefits of new treatments may sometimes be limited, other treatments may convey noteworthy health benefits. Under current Dutch policy, treatments that are clearly effective, such as CAR-T cell therapies, are also placed in the Coverage Lock. In March 2023, for the first time an effective treatment was not included in the basic healthcare package after the Coverage Lock procedure was finished, due to unsuccessful price negotiations. Trodelvy, a third-line treatment for triple-negative breast cancer which gives approximately 5.4 months life-prolongation and costs 68,707 euros per patient per treatment, was not included in the basic healthcare package (Rijksoverheid 2023). However, for some patients, also an estimated life-prolongation of 5.4 months could be valuable, for instance if an important life event is expected to take place in the life of the patient in these months. In such cases, starting with a new treatment such as Trodelvy, even if the expected health benefit is only a few months, might improve patients’ well-being.

While not all newly approved anti-cancer treatments are (highly) effective, beneficence does serve as a valid argument for prescribing effective non-reimbursed treatments, even if patients must pay out-of-pocket.

### Liberty and Autonomy

Another argument in favour of allowing people to pay for new treatments while these are not publicly funded, is the freedom of individuals to spend their private money as they see fit. In liberal democracies such as the Netherlands, citizens are—or should be—free to spend their after-tax income on whatever products or services they desire, including products or services that may promote their health. If physicians, hospitals, or health authorities prohibit patients from spending private money on (non-reimbursed) medical treatments, they infringe upon those patients’ rights to liberty and self-determination. Following the “harm principle” of nineteenth century philosopher John Stuart Mill—i.e. the principle that “the only purpose for which power can be rightfully exercised over any member of a civilized community, against his will, is to prevent harm to others” (Mill 1859)—they would only be allowed to do so if out-of-pocket payments were harmful to others. In the section on non-maleficence below, we will discuss the harms associated with allowing out-of-pocket payments.

In medical practice, the principle of respect for autonomy has been operationalized in the norm of informed consent: for every medical examination or treatment, patients must give their explicit consent. To be able to make an autonomous decision and provide informed consent, according to Beauchamp and Childress, three conditions must be met: intentionality, understanding, and non-control (Beauchamp and Childress 1979). Patients should have the intention of making the decision, understand their options, and be able to choose voluntarily, free from pressure or coercion. This implies, among other things, that patients should be well-informed about relevant treatment options, the expected benefits (or lack of them), and the possible side effects. The question here is whether non-reimbursed treatment options are considered to be “relevant treatment options” that should be offered to patients, and thus, about which patients should be informed as part of the informed consent process. In the Netherlands, currently, not all physicians inform patients regarding non-reimbursed treatments (Bomhof et al. 2022).

Emanuel and Emanuel distinguish four “models” for decision-making within the physician–patient relationship that could help to shed light on whether non-reimbursed treatments should be discussed with patients; the paternalistic model, the informative model, the interpretive model, and the deliberative model (Emanuel and Emanuel 1992). In the paternalistic model, the physician takes a directive stance and chooses *for* the patient to improve his or her well-being. It would be paternalistic for physicians, for instance, to withhold information about non-reimbursed treatments from patients, or to stop patients from accessing such treatments, if they want to, in order to protect their well-being. In this case: to protect them from incurring impossible or debilitating financial costs, e.g. those of CAR-T cell therapies. This model clearly runs counter to the principle of respect for autonomy. By contrast, in the informative model, the physician simply offers information about possible treatment options, refrains from making any value judgments, and lets the patient decide. For instance, the physician would inform all patients about all relevant non-reimbursed treatment options, regardless of their ability to pay, and regardless of whether or not these options, in practice, may be feasible—whether or not, for instance, the hospital allows and makes it possible for patients to pay for medical treatments. The informative model expresses a “thin concept” of autonomy, but it is questionable whether it really enhances patients’ autonomy if it implies offering them “options” that are wholly beyond reach (and thus no real options). Neither the paternalistic nor the informative model will be preferable as a model for informed consent regarding non-reimbursed treatments.

Both the interpretive model and the deliberative model are more fitting with “thick concepts” of autonomy and put the patient’s personal values centre stage. In the interpretive model, the physician tries to understand the patient’s personal values and wishes, and possibly also the patient’s financial situation, and may discuss (only those) treatment options that are relevant and feasible for the individual patient. In this model, the physician might differentiate between patients who do and who do not have the financial means to access non-reimbursed treatments and inform only those patients who do have the financial means *and* the wish obtain the treatment based on their personal values. In the deliberative model, finally, the physician enters into a critical discussion with the patient on the latter’s personal values, and together they decide which values and goals are suitable given the medical situation of the patient, and which treatment plan is fitting. Both models leave room for the physician’s interpretation of whether a non-reimbursed treatment would be a relevant or fitting option for a patient, and whether it should be brought up in the consultation room.

It follows from our analysis of the principle of respect for autonomy that physicians should inform patients about relevant non-reimbursed treatment options and allow patients to pay for them, if these treatments align with patients’ personal values and goals *and* if these treatments are in fact accessible for patients. Presupposing that patients have sufficient decision-making capacity and are able to make decisions freely, it would be paternalistic not to inform them to protect their well-being. However, informing patients about non-reimbursed treatment options that are (wholly) inaccessible, for instance, because they are extraordinarily expensive, may not enhance patients’ autonomy, either. To respect patients’ autonomy, physicians may need to enquire into patients’ personal values and goals, and discuss with patients whether they want to be offered—information about—medical treatments that are not (yet) reimbursed.

## Ethical Arguments Against Out-of-Pocket Payment

There are two important arguments against allowing out-of-pocket payment for expensive anti-cancer treatments. Because these arguments may be less self-evident, they merit more extensive analysis, in order to understand the *dilemma* of allowing out-of-pocket payments within universal healthcare systems.

### Non-maleficence

A first reason not to allow patients to pay for treatments out of pocket is to protect them against potential harms. This is in line with the principle of non-maleficence (Beauchamp and Childress 1979), which refers to the Latin adage “primum non nocere,” which means “above all, do no harm” (Smith 2005). This principle was part of the classical Hippocratic Oath, as reflected in the phrase: “*I will keep them [the ill] from harm and injustice*’’ (Askitopoulou and Vgontzas 2018).

In the context of expensive anti-cancer treatments, various types of harm to the individual patient can be identified. Firstly, there is a risk of potentially harmful side effects of the treatment. As said, however, this article focuses on anti-cancer treatments which are approved for marketing by the relevant drug regulatory authorities, and thus, are proven safe and (at least minimally) effective, and the potential for harmful side effects is no different from other (reimbursed) medical treatments. Thus, any potential for harm due to side effects does not serve as a valid counterargument against out-of-pocket payments. Secondly, out-of-pocket payments could have serious financial consequences for patients. This type of harm—referred to as “financial toxicity,”—by contrast, *does* occur uniquely in a context in which patients fund medical treatments themselves, and may need to make sacrifices to do so. Some patients may put themselves in debt or sell their house to be able to pay for treatments. A questionnaire-based study in the United Kingdom, for instance, showed that among patients and members of the general public, 22 per cent would remortgage their house to pay for medical treatments (Jenkins et al. 2011). Financial toxicity is associated with lower quality of life, stress, and anxiety (Arastu et al. 2020). Some patients are more at risk for financial toxicity than others. An Israeli study showed that the risk of financial burden of out-of-pocket spending was lower for patients with an above-average income or better education (Tur-Sinai et al. 2022). Not all patients may succeed in amassing the funds, leading to “subjective financial distress” (Carrera, Kantarjian, and Blinder 2018). Although we could not find any literature on whether and to what extent patients or their family members would suffer from financial toxicity if out-of-pocket payments were allowed in universal healthcare systems, we believe that, given the enormous costs of many new anti-cancer treatments, there is a significant probability that they might. Disallowing out-of-pocket payment would help prevent individual patients against financial burdens and hardship.

Also, allowing out-of-pocket payments in a universal healthcare system might possibly be harmful to other patients. If it is, the harm principle may come into play. For other patients, healthcare professionals, and possibly citizens in egalitarian healthcare systems, being confronted with unequal opportunities to access healthcare—especially when this occurs within the same hospital or region—may have adverse impact on psychological well-being (Weale and Clark 2010). In the literature, negative psychological effects have been described of allowing top-up fees or private payments for cancer care in countries with two-tier healthcare systems such as New Zealand and the United Kingdom (Fenton 2011; Sikora and James 2009; Bloor 2008; Gubb 2008; Richards M. 2008). If allowing out-of-pocket payments were to harm others, psychologically, it would run counter to the principle of non-maleficence.

Other others-affecting types of harm occur on a macro-economic level. The first is displacement of healthcare. This might occur, for instance, if for the administration of treatments and for monitoring and follow up of any adverse events, patients need to make use of public hospital beds, equipment, and staff. While patients who pay out of pocket could also be charged for the ancillary costs associated with these services, in the context of increasing *absolute* scarcity in healthcare, they might usurp facilities and staff that, as a result, would no longer be available for other patients who rely on the publicly funded healthcare system. As this might not only harm other patients but also lead to unjust distribution of resources, displacement of care could both be seen as a problem of harm and a problem of justice. A recent study in the Netherlands suggests that displacement of care based on the introduction of new expensive technologies had not yet occurred (Wammes et al. 2020). However, little is known about whether out-of-pocket payments or top-up fees would lead to displacement of care in universal healthcare systems.* If* out-of-pocket payments were to lead to displacement of care, allowing them would be undesirable, as it would hinder the accessibility of care for other patients, which would be harmful to them.

The second is the potential for allowing out-of-pocket payments to undermine the bargaining power of governments. If pharmaceutical companies can tap into private markets, they may not be incentivized to lower their prices or fulfil the cost-effectiveness requirements of publicly funded systems, and may not be included in the basic package of healthcare that is accessible for all patients. If out-of-pocket payments were thus to lead to higher drug prices, governments would need to restrict inclusion of newly approved treatments in the basic package even further, to increase the premiums of mandatory health insurance or to cut down on healthcare generally, potentially lowering the quality of basic healthcare, all of which would negatively affect all citizens. However, allowing out-of-pocket payments might also have the opposite effect; if it were to contribute to the affordability of the healthcare system, as some treatments would then not have to be reimbursed collectively, and if it were to leave more funds available for other, more cost-effective treatments, this could potentially benefit all citizens, including the worst-off. To our knowledge, none of these effects—specific to (dis)allowing out-of-pocket payments—have been reported in the literature. Consequently, it is currently not clear whether allowing out-of-pocket payment has adverse effects on the scope or quality of healthcare provided within publicly funded healthcare systems.

Health policies should not be harmful to individual patients nor lead to harm to others. In order to determine whether allowing out-of-pocket payments in publicly funded healthcare systems is harmful—through financial toxicity or psychological burdens, or by displacing care or depleting the healthcare system—further empirical research is needed.

### Justice

A second main argument against allowing patients to pay for treatments out of pocket, is that it could potentially lead to unequal access to medical treatments among patients. Universal healthcare systems are based on the notion that every citizen should have equal access to healthcare based on medical need only, and regardless of their financial means, social status or other contingent factors. This commitment to ensure equal access to healthcare for every citizen follows from the principle of justice (Beauchamp and Childress 1979), which implies that health resources should be fairly distributed. However, there is no consensus on what fair distribution is. According to Norman Daniels, who has translated Rawls’s justice theory to the medical domain, healthcare should be distributed in such a way, that it promotes equal opportunities to participate in society (Daniels 2001). In a context of scarcity, Daniels’s theory implies that patients who need care the most in order to enjoy a normal range of opportunities, should be prioritized over others. Also, it implies that government spending on healthcare should be curtailed, so that other preconditions for having opportunities in life, such as education and safety, can be met. It follows that it is just for publicly funded healthcare systems not to reimburse treatments that are insufficiently cost-effective. However, Daniels’s theory does not explicitly address the question whether or not it is just for publicly funded healthcare systems to allow patients to pay out of pocket for non-reimbursed treatments.

There is only one set of theories of justice that does, namely: egalitarian theories of justice. Egalitarian theories of justice consider equality desirable and inequality undesirable in and of itself (Parfit 1997). These theories would point out that by allowing patients to pay for treatments themselves, a form of inequality is introduced: more affluent patients will have the opportunity to obtain access to non-reimbursed treatments, while less affluent patients will not. And if patients were to start crowdfunding campaigns to help shoulder the costs of non-reimbursed treatments, this would introduce further inequities within society (Snyder 2016). As long as the treatment cannot be made available to *all* patients, egalitarian theories go, it will be preferable to keep more affluent patients from obtaining access using private funds, even if this results in the treatment not being available at all. This phenomenon is known as “levelling down” (Eyal 2013), which means, in short, that if not everyone can get X, then no one should get X. Critics have argued against the fairness of this phenomenon, making the so-called levelling down objection (Parfit 1997). The objection entails that, if the worst-off (patients unable to pay out-of-pocket) are not becoming better off, but the well-being of the best-off (patients able to pay out-of-pocket) is reduced to the level of the worst-off, we have gained nothing good (Parfit 1997). For instance in the case of Trodelvy, it would be considered unjust to prevent patients from getting access to the drug and thereby deprive them of the chance of (on average) five months of life prolongation, just because not all patients can get access.

According to Rawls, there is one condition under which social inequalities can be morally acceptable, namely: this is when inequalities will benefit the citizens who have the least opportunities—those who are worst off. This he calls the “difference principle” (Rawls 1971). It follows from the difference principle that in order to determine whether or not allowing out-of-pocket payment for non-reimbursed treatment is just, it is important to assess whether it will or will not ultimately benefit the worst-off. Further empirical research will be needed to ascertain whether allowing out-of-pocket payments will lead to displacement of healthcare or depletion of the healthcare system or, by contrast, whether it will help relieve some of the financial pressures within the healthcare system and thus contribute to its sustainability, and thus will come to benefit the worst-off.

While according to egalitarian theories of justice, a policy of allowing out-of-pocket payments may not be morally desirable as it introduces inequalities in access to medical treatments, the theory of justice developed by Rawls and Daniels seems to leave room for allowing out-of-pocket payments as long as it may ultimately come to benefit the worst-off.

### Solidarity

Solidarity is another fundamental value underlying publicly funded healthcare systems. In the Netherlands, the National Health Care Institute, which advises the Ministry of Health, Welfare and Sports about which treatments to include in the basic health insurance package, describes its objectives on their website as follows:

“Every person residing or working in the Netherlands is entitled to good care services from the standard health insurance package. Our healthcare system is based on the principle of solidarity. Rich and poor, young and old, healthy and sick: everyone in the Netherlands has access to the same care.” (Zorginstituut Nederland 2021)

While the interpretation of solidarity may differ across European healthcare systems, solidarity is often seen as an important value (Saltman 2015).

The concept of solidarity is used in different ways. It can be seen as the promotion of equality—therefore sharing some similarities with egalitarian justice—but also as assisting patients in need, upholding a solidarity-based healthcare system or willingness to contribute (van Till et. al. 2023). Often, solidarity is used to refer either to a feeling, of “togetherness” or “unity,” or to a predisposition, a willingness to help people in need. Barbara Prainsack and Alena Buyx, who describe solidarity as a predisposition, define solidarity in the context of bioethics as follows: “solidarity signifies shared practices reflecting a collective commitment to carry ‘costs’ (financial, social, emotional or otherwise) to assist others” (Prainsack and Buyx 2012, 346). They distinguish three “tiers” on which solidarity becomes manifest: on the interpersonal level, in group practices, and on a societal level (Prainsack and Buyx 2012). All three of these tiers can be applied to out-of-pocket payments. Solidarity on an interpersonal level takes place between individuals, when people demonstrate a willingness to carry costs for others based on certain similarities. For instance, solidarity can be seen when people are willing to contribute financially to the non-reimbursed medical treatment of someone else through crowdfunding. On the second tier, solidarity becomes manifest within groups as a form of social behaviour based on a feeling of “being in the same boat.” Patients may (need to) choose to refrain from out-of-pocket payments for non-reimbursed medical treatments when they know that others, who are less affluent, will not be able to buy those same treatments. The third tier of solidarity takes place on a societal level and is expressed in contractual and legal arrangements. This is also seen in the Dutch healthcare system, in which everyone must contribute to the healthcare system by paying taxes and, in return, everyone has equal access to healthcare. On this tier, solidarity can thus be imposed by governments (Prainsack and Buyx 2012). Prainsack and Buyx also state that the third tier leans on the first two tiers. To be able to sustain a universal healthcare system based on solidarity, there has to be support for this within society and a feeling of togetherness amongst individuals. Thus, to be able to maintain such a healthcare system, the “willingness to carry costs” among citizens has to be safeguarded. This willingness to carry cost for the sake of others, within societies, will likely not be infinite. If citizens are obliged to make rising and very high (monthly) financial contributions to the universal healthcare system, the predisposition of solidarity (on tiers 1 and 2) could crumble. This is another reason (apart from justice) why governments should critically assess the cost-effectiveness of newly approved medical treatments and limit the basic healthcare package, to prevent mandated financial contributions from citizens from skyrocketing. Depending on the notion of solidarity used, solidarity may serve as an argument either for or against allowing out-of-pocket payments: on the one hand, helping or allowing patients to access non-reimbursed treatments can be seen as an expression of (tier 1) solidarity, namely, as a willingness to carry costs to help those in need. On the other hand, if solidarity is taken to mean “being in the same boat” (on the second tier), it may imply that affluent patients should refrain from pursuing access if other, less affluent patients, are not in the position to obtain the same benefits. Thus, out-of-pocket payments may not be conducive to sustaining a solidaristic healthcare system.

## Discussion

Out-of-pocket payments for expensive anti-cancer treatments pose an intractable ethical dilemma for countries with a universal healthcare system. While in countries with two-tier healthcare systems or large private sectors, private spending on healthcare is common and the moral values of liberty and beneficence take prominence, allowing out-of-pocket payments can at the same time jeopardize the values of non-maleficence, justice, and solidarity. This poses difficulties for countries in which the healthcare system is publicly funded and profoundly based on values of equality and solidarity, as shown in relation to the case-study of the Dutch healthcare system.

In this article, we have critically examined the ethical arguments both in favour of and against out-of-pocket payments. The aim of this paper, however, is not to take a normative stance in the discussion regarding out-of-pocket payments. Rather, it aims to show that the weighing of relevant arguments is highly dependent on contextual factors, such as the effectiveness of specific medical treatments vis-à-vis the potential harms of allowing out-of-pocket payments—including financial toxicity and any adverse consequences for the accessibility or quality of public healthcare through displacement of care. Furthermore, the socio-cultural context of the healthcare system, including the emphasis which is put within society on solidarity as a moral value, plays an important role in how these arguments are weighed. Therefore, we believe that the decision whether to allow out-of-pocket payments is ultimately a political decision. As both the decisions to allow or to disallow out-of-pocket payments have negative consequences, we believe there is an important role for rational democratic deliberation in the weighing of these arguments. Through rational democratic deliberation, citizens can bring to the fore specific societal values and reasonable arguments (Fleck 2012). The aim of such a rational democratic deliberation process is to work towards a shared understanding, and ensure a procedurally just decision-making process. In the context of healthcare allocation, ethicists have already emphasized the importance of democratic deliberation leading to procedurally just (and fair) outcomes (Gruskin and Daniels 2008; Daniels 2016). Through rational democratic deliberation, the decision whether to allow out-of-pocket payments can be tailored to the local socio-cultural context and adjusted to the moral values at stake in a given healthcare system. While we believe that whether to allow out-of-pocket payments ultimately remains a political decision, and is time- and place-dependent, this paper provides some important normative considerations for policymakers to take into account when designing policies for out-of-pocket payments, which we will discuss in-depth below.

Firstly, it is important to be aware that the arguments based on justice and solidarity are ambiguous. Justice can be a reason not to allow out-of-pocket payments, but this largely depends on the theory of justice which is being used. While egalitarian theories will consider all out-of-pocket payments unjust, as it increases inequality amongst affluent citizens and less affluent citizens, according to Rawlsian justice theories, (some level of) inequality may be acceptable, as long as it ultimately (also) benefits the worst off. Allowing out-of-pocket payments could benefit the worst off, for instance by alleviating pressure on the healthcare budget, if the introduction of a second tier would make it easier for healthcare systems to deny reimbursement of (almost) insufficiently cost-effective medical treatments. Thus, if it would make room for the provision of more cost-effective healthcare to more patients within the public healthcare system. In that case, the justice argument against allowing out-of-pocket payments loses part of its significance: it would not lead to the displacement of care and thus disadvantage others, but, instead, serve to help sustain the provision of decent cost-effective healthcare for the population. The same goes for solidarity. Allowing patients to pay for non-reimbursed medical treatments may adversely impact solidarity understood as the feeling of “being in the same boat” and consequently, it might undermine societal willingness to contribute financially to the solidary healthcare system. However, it might also do the reverse, if it serves to alleviate pressure on the healthcare budget and helps maintain the system.

It is clear that to be able to weigh these ethical arguments in a given healthcare system, additional empirical research on the consequences of allowing out-of-pocket payment will be needed. To assess whether the harm principle comes into play, evidence is needed on displacement of care, on the psychological and psychosocial effects of out-of-pocket payments on patients who cannot afford to do so and on healthcare professionals, and on the potential macro-economic and societal consequences of allowing out-of-pocket payments in a universal healthcare setting. In the United Kingdom, insurers provide additional insurance for top-up-fees in the NHS (Bupa 2022) As Desai and colleagues point out, it is important to monitor these practices for potential adverse consequences (Desai et al. 2009). Also, additional research is needed on the risks of financial toxicity for individual patients who are paying for non-reimbursed medical treatments. Without empirical evidence, it is not possible to properly assess the consequences of out-of-pocket payments.

Furthermore, if patients are allowed to pay for treatments using private funds, it is important that they are adequately informed about the (sometimes limited) expected effectiveness and the possible risks and side effects. Especially when patients are willing to make significant life alterations to be able to pay for a treatment, such as selling their house, it is important that they are adequately informed about the balance of risks and benefits. When great financial sacrifices would be required but the expected health benefits are limited, out-of-pocket payments might not be proportional. Studies show that physicians do not always inform patients about treatments which are not yet reimbursed (Bomhof et al. 2022; Thomson et al. 2006). However, patients may expect their doctors to inform them. A study amongst the Australian general public showed that the majority of respondents (91 per cent) wanted to be informed about non-reimbursed medical treatments, even if only 51 per cent was willing to pay for the treatment out of pocket (Mileshkin et al. 2009). It is not clear whether physicians *ought to* inform patients about non-reimbursed treatment options, especially when the likelihood of medical benefit is limited. Both the interpretative and the deliberative model of Emanuel and Emanuel (1992) leave room for physicians to inform patients about non-reimbursed treatments, while also explaining the potential risks, including financial toxicity, and stressing that certain options might not be proportional. Here lies a task for the medical profession, to develop guidelines on informing patients about non-reimbursed treatments and the potential financial consequences of using private funds to pay for medical treatments. Additional measures, such as financial coaching, might be necessary to help patients make well-informed, autonomous decisions to protect them from harm (principle of non-maleficence).

Lastly, allowing patients to pay out of pocket for medical treatments that are not offered through the universal healthcare systems amounts to the introduction of a two-tier healthcare system. Benjamin Krohmal and Ezekiel Emanuel have argued that an ethically acceptable tiered healthcare system should meet five criteria; 1) there should be a core benefits package that covers an adequate level of healthcare, 2) which is guaranteed to all citizens, and 3) attracts a sizable majority of the population to use. Furthermore, 4) purchases of market tier services should be made with after-tax money and not provide exemption from tax obligations, and 5) easy adjustment of the core benefits package should be possible in response to changes in technology, data about efficacy and demand (Krohmal and Emanuel 2009). If allowing out-of-pocket payments for non-reimbursed treatments were not to diminish the core benefits package, and affluent citizens continued to contribute to the universal system, it could be argued, it would not lead to harm to others, and would be compatible with the difference principle. However, whether it is fair to allow patients to pay for a treatment out-of-pocket and introduce a second tier—again—strongly depends on the consequences out-of-pocket payments would have for others, e.g. through displacement of care, and the weight which is placed on moral values like equality and solidarity within a given society.

As the many expensive anti-cancer treatments which are entering the market will continue to contribute to rising healthcare costs, the number of non-reimbursed treatments in the future is expected to grow. And as long as, in the Netherlands as well as in other universal healthcare systems, policies for access to non-reimbursed treatments remain absent, the dilemma of allowing patients to pay for treatments themselves is likely to continue to present itself in the consultation room. Individual doctors or hospital managers may decide differently about letting patients pay out of pocket for non-reimbursed treatments, which may increase inequalities. Therefore, there is a pressing need for fair policies for access to non-reimbursed treatments in universal healthcare systems. This requires, at minimum, a better understanding of the potential consequences of out-of-pocket payment and the moral values at stake. With this article, we aim to draw attention to the ethical dilemma of out-of-pocket payments in universal healthcare systems and to help guide policymakers in dealing with this dilemma by presenting and critically discussing relevant normative arguments for and against out-of-pocket payments.
